# 

**Published:** 1881-12

**Authors:** 


### CONTENTS FOR DEC. 1881.

ORIGINAL COMMUNICATIONS.

ART I.—Abortion with Retained Placenta. By Wilmer Brinton, M. D., Baltimore 731

ORIGINAL TRANSLATIONS AND ABSTRACTS.

The Prevention of Ophthalmia of the New Born .......... 742

Contribution to the Study of Inherited Syphilis 744

Nitro-Glycerine in Migraine, Epilepsy and Anæmic Headaches .......... 750

Extral Genital Chancre .......... 750

Palliative Treatment of Atresia of the Vagina and Uterus .......... 751

Proper Treatment of Common Eye Diseases. 752

Fracture of the Neck of the Femur .......... 754

EDITORIAL DEPARTMENT.

The General and Special Education of Dentists .......... 758

Ourselves .......... 759

Gratuitous Dental Service in the French Primary School .......... 760

Dr. O. W. Wight .......... 760

BIBLIOGRAPHICAL DEPARTMENT .......... 771, 772

SELECTIONS.

On the Nature and Mode of origin of the Lead-Line in the Gums .......... 763
Aids to Diagnosis in Nasal Disease .......... 768
Removal of the Uterus for Cancer .......... 771
Stertorous Breathing in Apoplexy .......... 772
A Case of Suicide by Dynamite .......... 774
Ocular Symptoms in Different Diseases .......... 775
The Modern Remedies for Pertussis .......... 776
Nervous Symptoms of Lithæmia .......... 777
Why does Labor Come on? .......... 778
Milk as an Article of Diet .......... 779
Injection Brou .......... 779
Painful Neuroma of the Skin .......... 780
Syphilis in Different Countries .......... 780
The Curability of Uterine Displacements .......... 781
Peptonate of Mercury .......... 781
Absence of Part of the Diaphragm .......... 782
Ethylate of Soda as a Caustic .......... 782
Tonga .......... 782
Treatment of Gonorrhœal Conjunctvitis .......... 783
Deaths From Ether .......... 783

POPULAR SCIENCE DEPARTMENT.

Value of Experiment in Preventive Medicine 784
Raindrops, Hailstones and Snowflakes .......... 796
A Possible Preventive of Rabies .......... 799
Is Opium-Eating Injurious? .......... 800
Sewage in Oysters .......... 801
Small-Pox and Anti-Vaccinators .......... 801
Longevity of Brain-Workers .......... 802
NECROLOGICAL .......... 802

